# Natural Compounds for the Management of Parkinson's Disease and Attention-Deficit/Hyperactivity Disorder

**DOI:** 10.1155/2018/4067597

**Published:** 2018-11-22

**Authors:** Juan Carlos Corona

**Affiliations:** Laboratory of Neurosciences, Hospital Infantil de México Federico Gómez, Mexico

## Abstract

Parkinson's disease (PD) is the second most common neurodegenerative disorder with an unknown aetiology. The pathogenic mechanisms include oxidative stress, mitochondrial dysfunction, protein dysfunction, inflammation, autophagy, apoptosis, and abnormal deposition of *α*-synuclein. Currently, the existing pharmacological treatments for PD cannot improve fundamentally the degenerative process of dopaminergic neurons and have numerous side effects. On the other hand, attention-deficit/hyperactivity disorder (ADHD) is the most common neurodevelopmental disorder of childhood and is characterised by hyperactivity, impulsivity, and inattention. The aetiology of ADHD remains unknown, although it has been suggested that its pathophysiology involves abnormalities in several brain regions, disturbances of the catecholaminergic pathway, and oxidative stress. Psychostimulants and nonpsychostimulants are the drugs prescribed for the treatment of ADHD; however, they have been associated with increased risk of substance use and have several side effects. Today, there are very few tools available to prevent or to counteract the progression of such neurological disorders. Thus, therapeutic approaches with high efficiency and fewer side effects are needed. This review presents a brief overview of the two neurological disorders and their current treatments, followed by a discussion of the natural compounds which have been studied as therapeutic agents and the mechanisms underlying the beneficial effects, in particular, the decrease in oxidative stress.

## 1. Introduction

For many years, natural compounds have provided an efficient resource for the discovery of potential therapeutic agents. Among them, many natural products possess antioxidative, antiapoptotic and anti-inflammatory activities. In this review, we summarize the role of natural compounds and their therapeutic use for the management of dopamine-related diseases, divided into part 1 for Parkinson's disease and part 2 for attention-deficit/hyperactivity disorder. In addition, the current knowledge of the mechanisms underlying the potential beneficial effects of diverse natural compounds capable of counteracting the progression of this type of diseases is reviewed, as demonstrated by their antioxidative effects.

## 2. Parkinson's Disease

Parkinson's disease (PD) is the second most common progressive and chronic neurodegenerative disorder, characterised by the progressive loss of dopaminergic neurons in the substantia nigra (SN) and their projections to the striatum. Thus, the function of the nigrostriatal pathway becomes reduced and causes the development of movement disorder [1]. The basic characteristics of PD include tremor, rigidity, bradykinesia, and impaired balance; depression is also present in patients, affecting the quality of life. One of the pathological features of PD is the presence of Lewy bodies, which are intraneuronal proteinaceous cytoplasmic inclusions and include *α*-synuclein, ubiquitin, and neurofilaments found in all affected brain regions [2]. The pathogenic mechanisms of PD include oxidative stress, mitochondrial dysfunction, protein dysfunction, inflammation, autophagy, and apoptosis [3]. PD occurs 95 % as a sporadic form, while familial forms involve mutations in proteins that include PINK1, DJ-1, PARKIN, FBXO7, and LRRK2 [4], even though pesticides, chemicals, and metals may increase the risk of developing PD. Currently, the treatment of PD includes drugs such as L-DOPA, which is catalysed primarily by dopa decarboxylase in the brain and is converted into dopamine, producing its therapeutic effects. Another treatment includes anticholinergic drugs that can block striatal cholinergic receptors inhibiting the excitability of cholinergic nerves; it has also been demonstrated that they can inhibit dopamine reuptake to enhance the function of dopaminergic neurons [5]. At present, there are also other drugs in Phase III clinical trials. Nevertheless, the current drugs used for the PD treatment have some side effects, limiting their clinical applications [6]. Drugs used for the PD treatment and their side effects are shown in Table 1. Thus, the growing interest in alternative therapies for neurodegenerative disorders, including PD, has focused on the neuroprotective and antioxidant effects of natural products that may provide alternatives, since they can have high efficiency and fewer side effects. Natural compounds used as alternative therapies for the management of PD are shown in Table 2.

### 2.1. Ginkgo biloba

Ginkgo biloba is an ancient tree native to China and has been extensively used in traditional Chinese medicine to manage symptoms associated with dysfunctions of the heart and lungs. G. biloba usually contains three ingredients, which include flavonoids, terpenoids, and ginkgolic acid [81]. Ginkgolides well-known plant extracts obtained from leaves from G. biloba, especially in the preparation EGb761, which contains ginkgolide B and bilobalide, have emerged as natural therapeutic compounds, in part due to their antioxidant activity. These effects have been observed in the 1-methyl-4-phenyl-1,2,3,6-tetrahydropyridine- (MPTP-) treated mouse model of PD, where chronic ingestion of EGb761 prevented MPTP-induced reduction in the dopaminergic nerve endings [9]. In addition, EGb761 administered before or after MPTP treatment protected against MPTP-induced dopaminergic neurotoxicity [10]. Moreover, EGb761 attenuated the neurotoxic effect of levodopa in the 6-hydroxydopamine (6-OHDA) model of PD, indicated that levodopa is neurotoxic and that EGb761 may decrease this toxicity [11]. The neuroprotective effects of EGb761 were demonstrated in the 6-OHDA rat model, as indicated by the reduction in the behavioural deficit in the rat [12]. Paraquat is a pesticide that has been linked to PD, and it has been demonstrated that EGb761 protects against paraquat-induced apoptosis of PC12 cells by increasing bcl-2 activation, maintaining of mitochondrial membrane potential (∆Ψ_m_) and decreasing caspase-3 activation through the mitochondria-dependent pathway [13]. The neuroprotective effect of EGb761 against MPTP neurotoxicity is associated with the blockade of lipid peroxidation, reduction of oxidative stress, and attenuation of MPTP-induced neurodegeneration of the nigrostriatal pathway [14]. Also, it was demonstrated in an extensive review that EGb761 may exert therapeutic actions in an animal model of PD via the antioxidant effects [82]. Ginkgetin, a natural biflavonoid isolated from leaves of G. biloba, protected against 1-methyl-4-phenylpyridinium ion- (MPP^+^-) induced cell damage in vitro by decreasing the levels of intracellular reactive oxygen species (ROS) and by maintaining ∆Ψ_m_ and also improved the sensorimotor coordination in a mouse PD model induced by MPTP, suggesting that the neuroprotective mechanism of ginkgetin occurs via regulating iron homeostasis [16]. In low-dose whole-body *γ*-irradiation in the reserpine model of PD, EGb761 was protective by ameliorating the reserpine-induced state of oxidative stress, mitochondrial dysfunction, and apoptosis in the brain [15]. The pretreatment with ginkgolide B or bilobalide protected SH-SY5Y cells against *α*-synuclein-induced cell injury and apoptosis [17]. Ultimately, the G. biloba extract treatment improved locomotor activity, decreased oxidative damage, maintained the dopamine homeostasis, and inhibited the development of PD in A53T *α*-synuclein transgenic mice [18].

### 2.2. Ginseng

Ginseng is a traditional Chinese herb containing more than 30 ginsenosides, the active ingredients of ginseng. Ginsenosides Rb1 and Rg1 are regarded as the main compounds responsible for the therapeutic actions of ginseng. Previous studies have shown that, in SN-K-SH cells, both ginsenosides Rb1 and Rg1 reversed MPTP-induced cell death [19]. The protective effect of Rg1 against MPTP-induced apoptosis was attributed to enhancing Bcl-2 and Bcl-xl expression, reducing Bax and iNOS expression, and inhibiting activation of caspase-3 [20]. The ginseng extract G115 significantly blocked tyrosine hydroxylase- (TH-) positive cell loss in the SN and reduced the appearance of locomotor dysfunction in two rodent models of PD [21]. Pretreatments of Rg1 or N-acetylcysteine were found to protect against MPTP-induced SN neuron loss by preventing glutathione (GSH) reduction, attenuate the phosphorylations of JNK and c-Jun, and activate superoxide dismutase (SOD) [22]. MPP^+^-induced cytotoxicity in SH-SY5Y was inhibited by water extract of ginseng, as demonstrated by the inhibitory effect on cell death, overproduction of ROS, elevated Bax/Bcl-2 ratio, release of cytochrome c, and activation of caspase-3 expression [23]. It was demonstrated that ginsenosides protect by reducing intracellular ROS levels, enhancing antioxidant activity, preserving the activity of complex I, stabilising the ∆ Ψ_m_, and increasing intracellular ATP levels. Rg1 treatment restored motor functions in MPTP-treated mice and these behavioural ameliorations were accompanied by restoration of dopaminergic neurons in the SN and striatum [24]. Additionally, the ginsenoside Rb1 exhibited a strong ability to disaggregate fibrils and to inhibit the polymerisation of *α*-synuclein [25]. Rg1 treatment inhibited the activation of microglia and reduced the infiltration of CD3+ and T cells and also protected TH-positive cells in the SN and reduced the serum concentrations of proinflammatory cytokines TNF*α*, IFN*γ*, IL-1*β*, and IL-6 in MPTP mouse models [26]. Also, using in vivo and in vitro models of PD, it was demonstrated that ginsenoside Rg1 may exert therapeutic effects on PD via the Wnt/*β*-catenin signalling pathway [27]. In the MPTP-probenecid mouse model, the oral treatment with Rg1 significantly attenuated MPTP-induced mortality, behaviour defects, and loss of dopaminergic neurons. The protective effect of Rg1 may be mediated by reducing aberrant *α*-synuclein-mediated neuroinflammation [28]. The neurotoxin rotenone is an inhibitor of complex I and has been widely used in vivo and in vitro to model PD. Thus, cotreatment with ginsenosides Rd and Re inhibited the increased intracellular ROS production and lipid peroxidation accumulation caused by rotenone. Besides, the major ginsenosides Rd and Re upregulate SOD and aconitase activities, and GSH also attenuates the depolarisation of ∆ Ψ_m_ and restores Ca^2+^ levels and moreover prevents apoptosis by modulating Bax and Bcl-2 and inhibiting cytochrome c release and caspase-3 activation [29]. Additionally, ginsenoside Rd reversed the loss of TH-positive cells in SN in vivo and in vitro PD models, which may involve its antioxidant effects and mitochondrial function preservation [30].

### 2.3. Flavonoids

Flavonoids are part of a large group of natural polyphenol phytochemicals with a long history of use as therapeutic agents. Baicalin, a flavonoid isolated from* Scutellaria baicalensis*, is the main metabolite of baicalein. The neuroprotective efficacy of baicalein has been shown in an in vivo model of PD using the neurotoxin 6-OHDA, where the protective effects on dopaminergic dysfunction and lipid peroxidation were seen [31]. Other reports showed that baicalein prevented abnormal behaviour by increasing dopaminergic neurons and dopamine and serotonin levels in the striatum and also inhibited oxidative stress and astroglial response [32]. Luteolin and apigenin are flavones with similar structure; luteolin is found in celery, broccoli, parsley, thyme, and olive oil, and apigenin is present in vegetables, several fruits, and herbs. Luteolin protects dopaminergic neurons against inflammation-induced neurotoxicity by inhibiting microglial activation [41]. Moreover, baicalein exerts protective effects in vivo and in vitro against 6-OHDA [33]. In addition, baicalein protects cells against the toxicity of a point mutation in *α*-synuclein [34]. Baicalein also inhibited the formation of *α*-synuclein oligomers and consequently prevents its oligomerisation [35]. Quercetin is the aglycone form of a number of other flavonoid glycosides, such as rutin and quercitrin, and is found in citrus fruit, onions, and grains. In the 6-OHDA rat model, treatment of quercetin increased levels of antioxidant and striatal dopamine and reduced dopaminergic neuronal loss [44]. Kaempferol is a natural flavonoid which has been found in grapefruit, and other plant sources. It improves motor coordination, raises striatal dopamine and its metabolite levels, increases SOD and GSH activity, and reduces the content of lipid peroxidation, also preventing the loss of TH-positive neurons induced by MPTP [48]. There is also evidence of neuroprotection by kaempferol by autophagy in SH-SY5Y cells and primary neurons against rotenone toxicity [49]. It has been demonstrated that rutin protects dopaminergic neurons against oxidative stress induced by 6-OHDA [50]. Furthermore, it has been shown that quercetin protects against oxidative stress and increases activities of glutathione peroxidase (GPx), SOD, ATPase, AchE, and dopamine depletion in MPTP-treated mice [45]. The bioflavonoid rutin inhibits 6-OHDA-induced neurotoxicity in PC12 cells by activation of SOD, catalase, GPx, and total GSH and by inhibition of lipid peroxidation [51]. Moreover, in a rotenone model, quercetin has been shown to upregulate mitochondrial complex I activity and increase catalase and SOD activity [46]. Mitochondrial dysfunction in SH-SY5Y cells and upregulation of DJ-1 protein expression induced by 6-OHDA is prevented by baicalein [36]. The flavonol isoquercitrin protects PC12 cells against 6-OHDA-induced oxidative stress [52]. Baicalein downregulates the activation of NF-*κ*B, ERK, and JNK and attenuates astrocyte activation in MPTP mice [37]. Baicalein inhibits the upregulation of proinflammatory cytokines in the SN and striatum in PD mice models [38]. Luteolin also reduces cytotoxicity induced by 6-OHDA and ROS production in neuronal PC12 cells by modulating changes in the stress response pathway [42]. In MPTP-treated mice, luteolin and apigenin protect dopaminergic neurons by reducing oxidative damage, neuroinflammation, and microglial activation and also improve muscular and locomotor activity [43]. The flavonoids and their metabolites can interact with neuronal receptors and modulate kinase signalling pathways, transcription factors, and gene and/or protein expression, which control memory and learning processes in the hippocampus [83]. Naringin is a flavonoid glycoside that is contained abundantly in the skin of grapefruit and orange and is the origin of their bitterness. It protects dopaminergic neurons by induction of the activation of mammalian target of rapamycin complex 1 (mTORC1) and inhibited microglial activation in the SN of the mouse treated with 6-OHDA [84]. In a rotenone mouse model, baicalein prevented the progression of *α*-synuclein accumulation and protected dopaminergic neurons and also inhibited the formation of *α*-synuclein oligomers [39]. It has been shown that baicalein inhibits *α*-synuclein aggregates and autophagy in rats treated with MPP^+^ [40]. Besides, in the same model, apigenin ameliorated dopaminergic neuronal loss and improved behavioural, biochemical, and mitochondrial enzyme activities; such effects were associated with the suppression of oxidative stress and neuroinflammation [53]. Recently, it was demonstrated that quercetin improved mitochondrial biogenesis and induced the activation of two major cell survival kinases, PKD1, and Akt and also enhanced CREB and BDNF (a CREB target gene) in MN9D cells against 6-OHDA-induced neurotoxicity, and in the MitoPark transgenic mouse model of PD, quercetin improved behavioural deficits and reduced TH cell loss [47]. Troxerutin (also known as vitamin P4) is a natural derivative of the bioflavonoid rutin that is present in coffee, cereal grains, tea, and vegetables. The neuroprotective effects of troxerutin were reported in a 6-OHDA rat model to reduce striatal lipid peroxidation, ROS, GFAP, and DNA fragmentation. Meanwhile, troxerutin was capable of preventing loss of TH-positive neurons in the SN [54]. The bioflavonoid hesperidin is a specific flavonoid glycoside frequently found in oranges and lemons. Hesperidin protects against iron-induced oxidative damage and dopamine depletion in Drosophila melanogaster model of PD [55]. In addition, in 6-OHDA-treated mice, hesperidin protects by reducing oxidative damage, increasing the dopamine levels and also improving the behavioural parameters [56].

### 2.4. Valeriana officinalis

Valeriana officinalis (Valerian) is a plant with sedative and antispasmodic effect, traditionally used in the treatment of insomnia, anxiety, and restlessness. The effects of valerian on rotenone-induced cell death in SH-SY5Y cells have been demonstrated [85]. Moreover, extract of valerian was effective in reducing the toxicity induced by rotenone in Drosophila melanogaster, as confirmed by the normalisation in the expression of SOD and catalase mRNAs, suggesting that the effects of valerian are, at least in part, associated with the antioxidant properties of the plant due to its phenolic and flavonoid constituents [57]. Valeriana wallichii, also known as Indian valerian or Tagar-Ganthoda, belongs to the family Valerianaceae and is considered as an important Asian counterpart of the European valerian. Thus, valeriana wallichii treatment significantly recuperated the altered behaviour, striatal dopamine levels, increased GFAP expression, and the histopathological changes observed in mice treated with MPTP. Likewise, it ameliorated the increased levels of ROS, inflammatory cytokines and lipid peroxidation and also ameliorated the diminished levels of antioxidants [58].

### 2.5. Passion Flower

Passion flower, commonly known as Passiflora incarnata (Passifloraceae), contains flavonoids, glycosides, alkaloids, and phenolic compounds. Also, it has been used for the treatment of anxiety, insomnia, epilepsy, muscular spasms, and other diseases [86]. Therefore, the biological effects of passion flower have been investigated in PD. The extract of passion flower reduced the number of jaw movements induced by tacrine, which is a widely used animal model of PD tremors. In addition, the model showed cognitive improvement, with significantly reduced duration of haloperidol-induced catalepsy. The passion flower possesses antioxidant activity, as shown by its significant scavenging ability [59]. Passiflora cincinnata is a Brazilian native species of passion flower and its possible biological effects have been investigated. Thus, in a model of PD induced by reserpine, Passiflora cincinnata extract prevented the decrease in TH in the SN induced by reserpine, delayed the onset of motor impairments, and prevented the occurrence of increased catalepsy behaviour. However, the extract did not modify reserpine-induced cognitive impairments [60].

### 2.6. St. John's Wort

The use of St. John's wort, known as Hypericum perforatum, dates back to the time of the ancient Greeks. Active compounds of St. John's wort have been identified and include naphthodianthrones, phloroglucinols, and flavonoids (such as phenylpropanes, flavonol glycosides, and biflavones), as well as essential oils. Therefore, the active compounds provide antioxidant and neuroprotective effects [87]. Two standardised extracts of St. John's wort have been tested on the neurodegeneration induced by chronic administration of rotenone in rats. Accordingly, St. John's wort reduced neuronal damage and inhibited the apoptotic cascade by decreasing Bax levels [61]. Besides, intrastriatal 6-OHDA-lesioned rats were treated with an the extract of St. John's wort and showed lowered striatal level of malondialdehyde, enhanced catalase activity, reduced GSH content, normalised expression of GFAP and TNF*α*, lowered DNA fragmentation and prevention of damage to dopaminergic neurons [62].

## 3. Attention-Deficit/Hyperactivity Disorder

Attention-deficit/hyperactivity disorder (ADHD) is the most common neurodevelopmental disorder in childhood and is characterised by inattention, impulsivity, and hyperactivity. The worldwide prevalence of ADHD in children and adolescents is 5.3% [88]; across cultures the prevalence is estimated to range from 5% to 7% [88, 89]. ADHD has high impact on school performance and causes impairments in personal, social, or occupational function, leading to isolation, poorer grades, and in adolescence there is increased risk of depression, delinquent and antisocial behaviour, incurring comorbid conditions, and later substance abuse [90]. ADHD has been associated with deregulation of the catecholaminergic pathway in the brain [91], although extensive data point to oxidative stress as potential contributor to the pathophysiology in ADHD [92]. Currently, ADHD treatment is pharmacologic and improves the attention, reducing distractibility and impulsive behaviour. Pharmacologic agents used for the treatment of ADHD increase levels of catecholamines in the brain, alleviating ADHD symptoms, and can be divided into two: psychostimulants and nonpsychostimulants [93]. Methylphenidate (MPH) is a psychostimulant that increases extracellular dopamine and norepinephrine levels, thereby correcting the underlying abnormalities in catecholaminergic functions and restoring neurotransmitter imbalance [94]. Atomoxetine (ATX) is a nonpsychostimulant, a norepinephrine specific reuptake inhibitor that increases catecholamine levels in the brain resulting in behavioural improvement [94, 95]. ADHD is a heterogeneous disorder and is categorized into three subtypes: hyperactive/impulsive, inattentive, and the combined subtype [89, 90]. There may be also differences in terms of treatment responses, for example, the hyperactive/impulsive subtype responds well to MPH, the inattentive subtype responds better to ATX, and the combined type responds well to ATX and MPH, although controversy still persists about which is the best treatment for each subtype. However, it has been demonstrated that the pharmacological treatments to improve ADHD symptoms have some side effects, which include nausea, headache, insomnia, abdominal pain, decreased appetite, and motor tics [96]. Drugs used for the ADHD treatment and their side effects are shown in Table 1. Moreover, the use of psychostimulant medications in ADHD has been also associated with increased risk of substance use disorder [97]. Thus, there has been growing interest in alternative treatments for ADHD, including natural compounds because of their antioxidant properties [98]. Natural compounds used as alternative therapies for the management of ADHD are shown in Table 3.

### 3.1. Ginkgo biloba

The extract from G. biloba leaves has been used as a herbal medicine for dementia [81, 99]. Therefore, several reports indicate that G. biloba may have therapeutic benefits in ADHD, given that the cotreatment with G. biloba and ginseng were found to alleviate ADHD symptoms in children, with minor side effects observed [63]. Moreover, in a double-blind, randomised trial the administration of G. biloba was less effective than MPH in the treatment of ADHD [64]. G. biloba extract treatment at a maximal dosage of 240 mg improved the behavioural ratings of ADHD symptoms and the electrical brain activity in children, suggesting that G. biloba extract seems to be well tolerated in the short term and may be a useful treatment [65]. Lately, in a randomised, placebo-controlled trial the response rate was higher with G. biloba treatment compared to placebo. Thus, G. biloba could be an effective complementary treatment for ADHD [66].

### 3.2. Ginseng

Ginseng contains a class of phytochemicals called ginsenosides, which are known as potent antioxidants and for their neuroprotective properties. Ginseng has been shown to alleviate effectively symptoms of ADHD. In an open trial, ginseng medication improved the inattention and hyperactive/impulsive score in children with ADHD [67]. Indeed, an observational clinical study showed that ginseng, given at 1000 mg for 8 weeks, improved inattentiveness in children with ADHD [68]. A double-blind randomised, placebo-controlled trial reported that ginseng decreased inattention and hyperactivity scores in children with ADHD [69]. Hence, ginseng has the potential to be used as an alternative therapy for ADHD. On the ADHD-like condition induced by Aroclor1254, YY162, which consists of terpenoid-strengthened G. biloba and ginsenoside Rg3, attenuated the increase in ROS and decrease in BDNF levels in SH-SY5Y cells. Moreover, YY162 attenuated reductions in p-TrkB, BDNF, dopamine transporter, and norepinephrine transporter expression [70].

### 3.3. Flavonoids

Pycnogenol is a herbal dietary supplement extracted from French maritime pine bark whose main ingredient is procyanidin. Procyanidins are members of the proanthocyanidin a class of flavonoids and are powerful antioxidants also found in food such as grapes, berries, pomegranates, red wine, and nuts. The treatment of 1-month pycnogenol administration resulted in a significant reduction in hyperactivity, improved attention, and visual-motor coordination and concentration in children with ADHD [71]. Moreover, pycnogenol reduced oxidative damage to DNA, normalised total antioxidant status, and improved attention, as demonstrated in a randomised, double-blind, placebo-controlled study [72]. Also, the administration of pycnogenol in children with ADHD, normalised catecholamine concentrations, leading to less hyperactivity, and reduced oxidative stress, in a randomised, double-blind, controlled design [73]. St. John's wort and pycnogenol have been tested as therapeutic alternatives to treat ADHD. A significant increase of SH-SY5Y cell survival was induced by pycnogenol, which did not cause any cytotoxic effect when used in therapeutically relevant concentrations; also, treatment with St. John's wort significantly increased ATP levels [100]. Oroxylin A is a flavonoid isolated from the root of Scutellaria baicalensis Georgi, a herb found in East Asia. It has been observed that oroxylin A is an antagonist of the GABA-A receptor and its neuroprotective actions include antioxidant, anti-inflammatory, and memory-enhancing effects. On the other hand, spontaneously hypertensive rats (SHRs) display some symptoms of ADHD, which makes them a model of the disorder. It was demonstrated that oroxylin A improved ADHD-like behaviours via enhancement of dopaminergic neurotransmission and not the modulation of the GABA pathway in the SHR [74]. Furthermore, an oroxylin A analogue reduced hyperactivity, sustained inattention, and impulsivity in the SHR [75]. Baicalin regulated the motor ability and learning and memory abilities in the SHR and thus controlled the core symptoms of ADHD [76]. Also, baicalin improved synaptosomal ATPase and LDH activities in the SHR, suggesting that baicalin exerts its therapeutic effect by upregulating the AC/cAMP/PKA signalling pathway [101]. A randomised, double-blind trial to investigate the therapeutic benefit of pycnogenol in ADHD patients is in progress [102].

### 3.4. Valeriana officinalis

The efficacy of valerian has been evaluated in a double-blind, placebo-controlled pilot study, where valerian showed improvement in ADHD symptoms, in particular, sustained inattention, anxiety and impulsivity, and/or hyperactivity [77]. The GABA- A receptors are the substrate for the anxiolytic action of valerenic acid, a major constituent of valerian root extracts [103]. GABA is the main inhibitory neurotransmitter in the CNS, and its deficiency causes anxiety, restlessness, and obsessive behaviour, symptoms often seen in ADHD. The European Medicine Agency deemed that root extracts of valerian could be used for the relief of mild nervous tension and sleep disorders. However, more research is required to support the efficacy of valerian in the treatment ADHD.

### 3.5. Passion Flower

The effect of passion flower in alleviating ADHD symptoms was tested in a randomised study. In addition, a tolerable side effect profile may be considered as one of the advantages of passion flower as compared with MPH [78]. It seems that the mechanism of action of passion flower is mediated via modulation of the GABA-A and GABA-B receptors and its effects on GABA uptake [104]. Although the passion flower has shown pharmacological activity in preclinical experiments, including sedative, anxiolytic, antitussive, antiasthmatic, and antidiabetic activities, its supposed efficacy does not appear to be adequately corroborated in the literature, since clinical studies often present methodologies and procedures with weaknesses [105].

### 3.6. St. John's Wort

St. John's wort produces its therapeutic effects by involving inhibition of the reuptake of dopamine, serotonin, and norepinephrine [106]. In a randomised controlled trial, the use of St. John's wort for the treatment of ADHD over the course of eight weeks did not improve symptoms [79]. However, a preliminary study reported that treatment with St. John's wort improved some symptoms in ADHD patients [80]. Because of findings that St. John's wort has no adverse effects, more studies are required to determine efficacy in the treatment of ADHD.

## 4. Conclusions

The natural compounds discussed in this review appear to be promising for the treatment of PD and ADHD. Therefore, these compounds can lay the foundation for a new therapeutic approach for the treatment of these disorders. Natural compounds are more easily accepted by patients, since they are considered healthier than synthetic drugs. Although the use of natural compounds for the neurological disorders has been considered as a safe approach, they are still far from being standard treatments, due to the lack of controlled clinical studies that could corroborate both their high efficacy and safety. Hence, better designed and more rigorous clinical trials are required before they can be established as therapeutic compounds. Neither PD nor ADHD, until today, have a therapeutic option capable of counteracting the progression of the disease, and natural compounds are often able to modulate the progression of this type of disorder, as demonstrated by their decreasing of oxidative stress.

## Figures and Tables

**Table 1:** Drugs used in PD and ADHD and their side effects.

**Drugs for PD [7]**	**Mechanism**	**Side effects**
L-DOPA and Carbidopa	A precursor to dopamine, combined with carbidopa, which blocks aromatic amino acid decarboxylase	Nausea, vomiting, insomnia, psychosis, hallucinations, hypotension, arrhythmia, somnolence, nightmares
Selegiline, Rasagiline, Safinamide	Monoamine oxidase B inhibitors (MAO-B); increase the synaptic availability of dopamine; safinamide also inhibits glutamate release	Abnormal movements, dizziness, nausea, vomiting, dry mouth, persistent diarrhoea, constipation, weight loss
Entacapone and Tolcapone	Catechol-O-methyltransferase inhibitors (COMT); Increase CNS L-DOPA bioavailability by decreasing peripheral L-DOPA metabolism	Dyskinesia, nausea, sleep disturbances, arterial hypotension, liver toxicity, vomiting, dizziness
Pramipexole, Ropinirole, Rotigotine, Apomorphine	Dopamine agonists; stimulate the action of dopamine at postsynaptic receptors	Nausea, dyskinesia, hallucinations, sleepiness, sleep attacks, confusion, ankle oedema, orthostatic hypotension, constipation
Trihexyphenidyl and Benztropine	Anticholinergics; block acetylcholine in the striatum	Dry mouth, urine retention, hallucinations, dry eyes, constipation, blurred vision, loss of memory, and confusion, somnolence, dizziness
Amantadine	Is an antagonist of the NMDA type glutamate receptor and also stimulates dopamine release	Oedema, hallucinations, insomnia, confusion

**Drugs For ADHD [8]**	**Mechanism**	**Side effects**

Methylphenidate	Inhibits reuptake of dopamine and norepinephrine	Anxiety, agitation, insomnia, decrease appetite, weight loss, irritability, hypertension, headache, stomach pain, numbness
Amphetamines	Inhibit reuptake of dopamine and norepinephrine	Decrease appetite, weight loss, irritability, hypertension, anxiety, agitation, nervousness, depression
Atomoxetine	Selective norepinephrine reuptake inhibitor	Dry mouth, sedation, fatigue, increased sweating, hypertension, decrease appetite, dizziness, drowsiness, insomnia, itching, impotence, unusually fast or irregular heartbeat, increased suicidality
Imipramine, Nortriptyline, Amitriptyline, Desipramine	Tricyclic antidepressants; Inhibit reuptake of dopamine and norepinephrine	Dry mouth, sweating abnormalities, drowsiness/sedation, increased suicidality
Clonidine and Guanfacine	Agonists of the *α*-2 and *α*-2A adrenergic receptors in the prefrontal cortex	Dry mouth, fatigue, dizziness, profound withdrawal effects

**Table 2:** Effects of natural compounds used as an alternative therapy for PD.

**Source**	**Compound**	**Action mechanism**	**Refs**
Ginkgo biloba	EGb761	Antioxidant, inhibited toxic effect of levodopa, inhibited MAO activity, ↓behavioural deficit, anti-apoptotic, maintain ΔΨ_m_, ↑bcl-2, ↓caspase-3, ↓lipid peroxidation, ↓oxidative stress	[9–15]
Ginkgo biloba	Ginkgetin	↓ROS, maintain ΔΨ_m_	[16]
Ginkgo biloba	Ginkgolide B and Bilobalide	↓*α*-synuclein, anti-apoptotic	[17]
Ginkgo biloba	Ginkgo biloba extract	↑SOD, ↑GHS, ↓malondialdehyde	[18]
Ginseng	Ginsenosides Rb1 and Rg1	Antioxidant, anti-apoptotic, ↑bcl-2, ↓Bax, ↓iNOS, ↓caspase-3, ↑SOD, ↑GHS, ↓*α* -synuclein fibrils, ↓TNF-*α*, ↓IFN-*γ*, ↓IL-1*β*, ↓IL-6, ↑Wnt-1, ↑*β* -catenin, ↑GSK-3*β*, maintain ΔΨ_m_, ↓cytochrome c release	[19–28]
Ginseng	Ginsenosides Rd and Re	Antioxidant, anti-apoptotic, improved mitochondrial function	[29, 30]
Flavonoid	Baicalein	↓lipid peroxidation, ↑serotonin, ↓dopamine levels, ↓oxidative stress, ↓*α*-synuclein aggregation, ↑DJ-1, ↓NF-*κ*B, ↓ERK, ↓JNK	[31–40]
Flavonoid	Luteolin	↓Inflammation, ↓ROS, ↓oxidative stress	[41–43]
Flavonoid	Quercetin	Antioxidant, ↑SOD, ↑GPx, ↓oxidative stress, ↑ATPase, ↑catalase, ↑complex I activity, ↑PKD1, ↑Akt, ↑CREB, ↑BDNF	[44–47]
Flavonoid	Kaempferol	↑SOD, ↑GHS, ↓lipid peroxidation,↑autophagy	[48, 49]
Flavonoid	Rutin	↓oxidative stress, ↑SOD, ↑GPx, ↓oxidative stress, ↓lipid peroxidation, ↑catalase	[50, 51]
Flavonoid	Isoquercitrin	↓oxidative stress	[52]
Flavonoid	Apigenin	↓Inflammation, ↓ROS	[43, 53]
Flavonoid	Troxerutin	↓lipid peroxidation, ↓ROS, ↓DNA fragmentation	[54]
Flavonoid	Hesperidin	Antioxidant	[55, 56]
Valeriana officinalis	Extract of valerian	Normalised SOD and catalase mRNAs	[57]
Valeriana wallichii	Valeriana wallichii	↓ROS, ↓Inflammation, ↓lipid peroxidation	[58]
Passiflora incarnata	Extract of passion flower	Antioxidant	[59]
Passiflora cincinnata	Passiflora cincinnata	Antioxidant	[60]
Hypericum perforatum	Extract of St. John's wort	Antioxidant, anti-apoptotic, ↑GHS, ↑catalase, ↓malondialdehyde, ↓DNA fragmentation	[61, 62]

**Table 3:** Effects of natural compounds used as an alternative therapy for ADHD.

**Compound**	**Outcome**	**Refs**
Ginkgo biloba	Alleviates ADHD symptoms in children and has minor side effects	[63]
Ginkgo biloba	Double blind, randomised trial; was less effective than MPH in children with ADHD	[64]
Ginkgo biloba	Improved behavioural ratings of ADHD symptoms and the electrical brain activity in children	[65]
Ginkgo biloba	Randomised, placebo-controlled trial; the response rate was higher	[66]
Ginseng	Open trial, improved the inattention and hyperactive/impulsive score in children with ADHD	[67]
Ginseng	Observational clinical study, improved inattentiveness in children with ADHD	[68]
Ginseng	Double-blind randomised placebo-controlled trial; ↓inattention and hyperactivity scores in children with ADHD	[69]
Ginsenoside Rg3 and Ginkgo biloba	↓ROS, ↑BDNF levels, ↑p-TrkB, ↑BDNF, ↑dopamine transporter, ↑norepinephrine transporter	[70]
Flavonoid	↓hyperactivity, improved attention and visual-motor coordination and concentration of children with ADHD	[71]
Flavonoid	Randomised, double-blind and placebo controlled study; normalised total antioxidant status, ↓oxidative damage to DNA and improved attention in children with ADHD	[72]
Flavonoid	Randomised, double-blind controlled design; ↓hyperactivity, normalised catecholamine concentrations and ↓oxidative stress in children with ADHD	[73]
Flavonoid	↑dopaminergic neurotransmission, improved synaptosomal ATPase, regulated motor ability, learning and memory, ↓hyperactivity, inattention and impulsivity in the SHR model	[74–76]
Valeriana officinalis	double-blind, placebo-controlled pilot study; improved ADHD symptoms	[77]
Passiflora incarnata	Randomised study, alleviated ADHD symptoms and has tolerable side effect	[78]
Hypericum perforatum	Randomised controlled trial; did not improve symptoms in children with ADHD	[79]
Hypericum perforatum	A preliminary study; improved some symptoms in ADHD patients	[80]
